# Personal Metabolomics: A Global Challenge

**DOI:** 10.3390/metabo11110715

**Published:** 2021-10-20

**Authors:** Petr G. Lokhov, Oxana P. Trifonova, Dmitry L. Maslov, Steven Lichtenberg, Elena E. Balashova

**Affiliations:** 1 Analytical Branch, Institute of Biomedical Chemistry, 10 Building 8, Pogodinskaya Street, 119121 Moscow, Russia; oxana.trifonova@gmail.com (O.P.T.); dlmaslov@mail.ru (D.L.M.); sl@metabometrics.com (S.L.); balashlen@mail.ru (E.E.B.); 2 Metabometrics, Inc., 651 N Broad Street, Suite 205 #1370, Middletown, DE 19709, USA

**Keywords:** metabolomics, personal analysis, laboratory-developed test, challenges, mass spectrometry, *p*-value, disease signature

## Abstract

Today, the introduction of metabolomics, like other omics sciences, into clinical practice as a personal omics test that realizes the perfect analytical capabilities of this science has become an important subject. The assembled data show that the metabolome of biosamples is a collection of highly informative and accurate signatures of virtually all diseases that are widespread in the population. However, we have not seen the emergence of personalized metabolomics in clinical practice. This article analyzes the causes of this problem. The complexity of personal metabolic data analysis and its incompatibility with widely accepted data treatment in metabolomics are shown. As a result, the impossibility of translating metabolic signatures accumulated in databases into a personal test is revealed. Problem-solving strategies that may radically change the situation and realize the analytical capabilities of metabolomics in medical laboratory practice are discussed.

## 1. Introduction

Following genomics and proteomics, the results of metabolomics testing for medical purposes are promising [1,2]. The Metabolomics Society has noted that the study of metabolism at the global or ‘omics’ level is a rapidly growing field that can profoundly impact medical practice. Today, doctors use only a tiny fraction of the information in the metabolome. They usually measure only a narrow subset of substances in the blood to assess health and disease. The Metabolomics Society has declared that “the narrow range of chemical analyses in current use by the medical community today will be replaced in the future by analyses that reveal a far more comprehensive metabolic signature. This signature is expected to describe global biochemical aberrations that reflect patterns of variance in states of wellness, more accurately describe specific diseases and their progression, and greatly aid in differential diagnosis” [3].

The metabolic data accumulated over the last decades make it possible to create a holistic test through panoramic measurement of metabolites in a biomaterial. Metabolite databases contain comprehensive information about disease signatures, metabolic pathways, abnormal concentrations of metabolites associated with different conditions, and descriptions of metabolite locations in organs, tissues, and even their subcellular localization (Table 1). Thus, the introduction of personal metabolomics, i.e., the use of high-throughput measurements of large sets of low-molecular-weight substances in biosamples with the subsequent use of the metabolic data available today for diagnostic purposes of a particular person, has long been actual [4,5,6,7,8,9].

The success of the implementation of metabolomics in laboratory diagnostics has become even more intriguing because metabolomics tests can be implemented in a direct-to-customer format. Mass spectrometry of metabolites, widespread in metabolomics, is compatible with dry blood spot (DBS) samples [10], allowing the collection of capillary blood unaided at home. Subsequent transportation of DBS samples to the laboratory by mail or specialized courier service can make metabolomic tests convenient for customers and available almost everywhere. The capabilities of bioinformatics data treatment can produce the metabolomics analysis results in a user-friendly format, making them acceptable to a wide range of clients.

The concept of multiple measurements from a small sample of blood is a long-standing and exciting idea for BioTech that has attracted colossal funding. For example, a startup company that claimed to develop a rapid analytical technique for a small amount of blood was valued at $10 billion before it collapsed [15]. In conjunction with the simplified regulatory rules for omics tests, for example, a laboratory-developed test (LDT) [16], in some cases, only a Clinical Laboratory Improvement Amendments (CLIA) laboratory is required; therefore, it is difficult to find a more attractive new direction in laboratory diagnostics. However, despite the evident potential, the only company that has made metabolomic tests available is Metabolon, Inc (Morrisville, NC, USA). In 2018, they announced the availability of the Meta UDx™ test for the diagnosis of rare and undiagnosed diseases in children and adults; this test has been analytically validated under the CLIA as an LDT [17].

**Figure 1:**
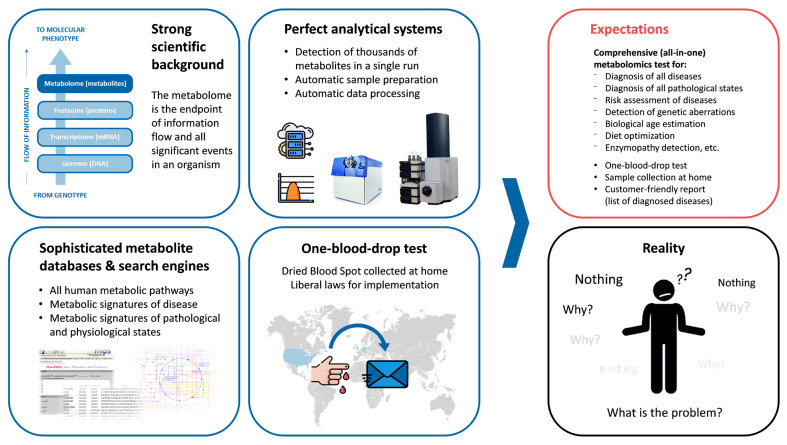
The mystery of translating metabolomics into personalized analysis.

What is the problem? Why is a relatively quick and comprehensive metabolomic analytical test not widespread in clinical practice (Figure 1)? The main aim of this paper is to highlight these problems, i.e., to show global challenges that prevent the entry of personal metabolomics into practical medicine inherent to metabolomics analytical and bioinformatic performance.

One of the reasons is the problem of standardization that occurs with multiple measurements. However, such difficulties in standardization are incomparable with the potential benefits of metabolomics-based diagnostics. In addition, there are already many standardization methods that can be implemented in high-tech laboratories, where blood samples, including those collected at home, can be sent from anywhere by mail as a DBS [10]. The next problem is the identification of metabolites, which is the bottleneck of all metabolomic studies. Metabolite identification is the most laborious and expensive part of the metabolomics analysis, which usually does not fit within the time and cost parameters of a personal test. Therefore, advances in this direction are also important for the introduction of personal metabolomics. Nevertheless, since these limitations are crucial for personal metabolomics, they are already well described [18] and not considered further in this article.

So, the boom in the introduction of such an attractive direction as personalized metabolomics is being held back by other more pronounced problems. Mass spectrometry laboratories that successfully work in metabolomics studies encounter them when switching to the analysis of a particular person. We also met a similar phenomenon in our practice. Our work in metabolomics research has been going on for many years. It has recently focused on personal data, which allowed us to see and describe the problems of personalized metabolomics.

It should also be noted that there are different technological platforms in metabolomics with their own advantages. The detection of large numbers of metabolites with high sensitivity is a significant advantage of mass spectrometry-based methods. Some reproducibility problems can be better addressed by nuclear magnetic resonance spectroscopy. However, the problems described below are independent of the analytical platform used, i.e., they are general to all personal metabolomics.

## 2. Main Challenges of Personal Metabolomics

### 2.1. First Challenge

The main problem is that metabolomic data are usually obtained from case–control studies. The design of such studies is very common in science. The group of control samples is compared with the experimental group (disease). In this case, all individual features that are not reproduced in the group are not statistically significant and thus are filtered out, and only statistically significant group-specific features are detected. For these types of studies, the t-test or the nonparametric Mann–Whitney–Wilcoxon test is usually used (Table 2). Such group-specific data are collected in the metabolite databases. Potential biomarkers are those metabolites whose levels in the sample demonstrate significant divergence from the norm (i.e., typically exhibit a high area under the receiver operating characteristic curve) and can be used in clinical laboratory tests. However, the emergence of new biomarkers of diseases today is a rare event. Specifically, the metabolites in the databases mainly provide biological insights and, due to weak diagnostic capacity, they may be used for diagnostics only together (as sets, molecular assemblies, signatures, footprints, molecular barcodes, etc.), summarizing their diagnostic potential. Unfortunately, this leads to the fact that the accumulated metabolomic data are not translated into a personal analysis, since the detection of metabolic signatures in personal data is challenging.

**Figure 2:**
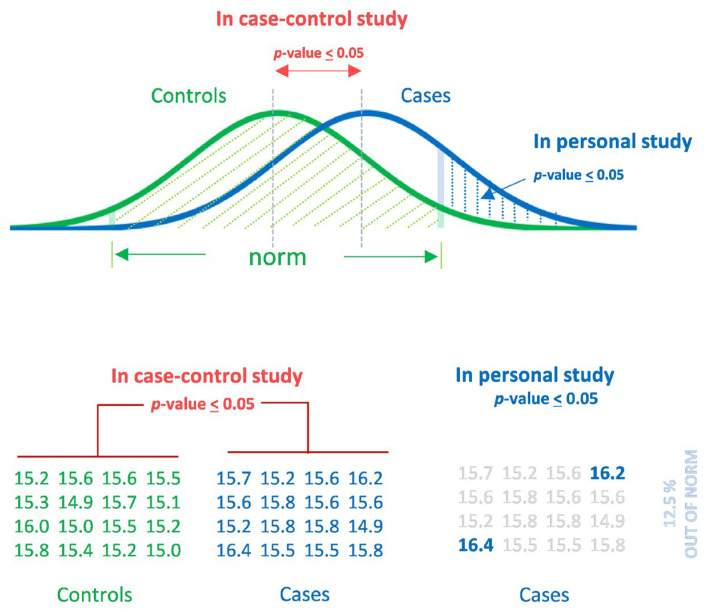
*p*-value-dependent challenge of personal metabolomics. A metabolite detected in a case–control metabolomic study as case-associated (*p* < 0.05) is not considered out of the norm in a personal metabolomics study. Case–control studies compare group characteristics. Individual (personal) *p*-values will overwhelmingly fit within the normal values (e.g., at *p* < 0.05, only 12.5% of cases will be outside of the normal range).

To understand this, let us look at the t-test. The t-test is a type of inferential statistic used to determine a significant difference between the means of two groups, which may be related to certain features. This implies that there are no prerequisites that a particular metabolite (i.e., not the mean of the group) in the signature should go beyond the norm. Rather, a significant part of the signature will be within the normal range. Figure 2 demonstrates this phenomenon for one metabolite with a *p*-value < 0.05 measured by the t-test (cases vs. control). Such a *p*-value is considered as a statistically significant difference between groups in biological science. However, in this example, only 12.5% of samples in the case group have a concentration of this metabolite outside of the norm. This indicates that metabolic signatures compiled by such metabolites should almost always give false-negative results for personal measurements.

It turns out that the generally accepted methods of data processing in metabolomics and the knowledge collected in the databases are not directly applicable in personal metabolomic tests because, in personal analysis, they most likely must correspond to the parameters of the norm. This is the crucial reason why the accumulated data of thousands of metabolomics studies and the technological excellence of measuring technology did not result in a revolution in laboratory diagnostics.

### 2.2. Second Challenge

**Figure 3:**
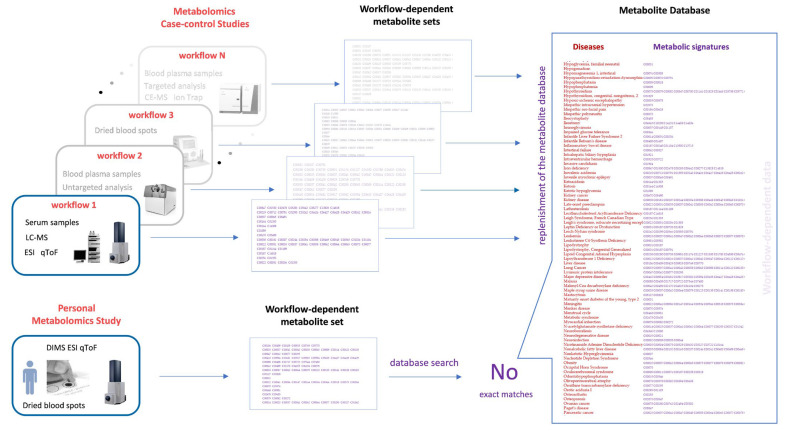
The workflow-dependent challenge of personal metabolomics. The sets of metabolites (including metabolic signatures of diseases) presented in the databases correspond to the protocols used for the metabolomic studies, which are diverse. Personal metabolic analysis is done according to its own protocol and cannot match all of the signatures obtained in various metabolomic studies and presented in the metabolite databases. Kyoto Encyclopedia of Genes and Genomes identifiers are shown for the metabolites. LC-MS, liquid chromatography-mass spectrometry; DIMS, direct-injection mass spectrometry; ESI, electrospray ionization; qToF, quadrupole time of flight; CE-MS, capillary electrophoresis-mass spectrometry.

The second problem lies in the fact that the metabolic data that fill the databases were obtained in various studies with different workflows (e.g., equipment and protocols). As illustrated in Figure 3, the set of metabolites measured in each case was specific for each study. Often, individual groups of metabolites are studied using targeted metabolomics, using highly sensitive detection of metabolites of a particular group (for example, using triple quadrupole mass spectrometers). Thus, the use of one personal test cannot cover the entire metabolite sets that were accumulated from a wide variety of metabolomics studies. Tuning for a particular set of metabolites to cover as much as possible inevitably leads to the deterioration of the detection of other sets. As a result, most metabolic signatures may only be fragmentarily detected in the personal analysis. Based on our experience, the coverage range for disease signatures is from 0% to about 30%. In terms of personal metabolomics, this looks like a very questionable result. Basically, for this reason, a panoramic metabolomics lab test, which is expected to have a high diagnostic coverage, quietly produces an array of false-negative results.

### 2.3. Third Challenge

**Figure 4:**
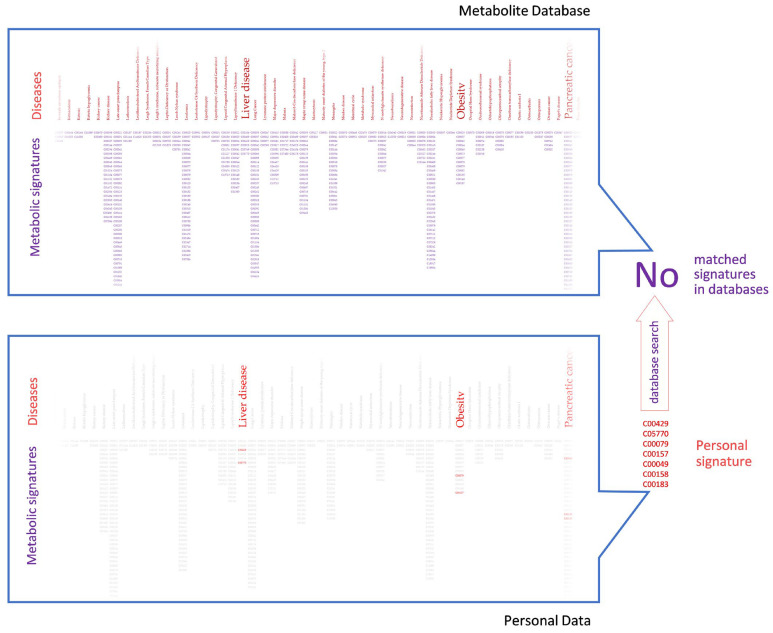
The problem of personal data searching against a metabolic database. The medley of fragmented signatures in personal data is searched against disease or other signatures in the database, leading to no matched signatures. Kyoto Encyclopedia of Genes and Genomes identifiers are shown for the metabolites.

The third challenge in itself may not be fatal; however, together with the other two challenges, it acquires an ominous character. As indicated earlier in classical metabolomics studies, group comparison allows the identification of metabolites associated with the disease under investigation. Such group-specific data, filtered out from individual ‘noise’, become statistically significant, can be projected onto relevant disease metabolic pathways, and can be successfully treated by metabolite set enrichment analysis [19,20]. In personal analysis, such extremely effective filtering is not possible. The entire set of metabolites supposedly different from the norm is associated with various diseases, pathological conditions, individual characteristics, etc., and is subject to interpretation. The database search of such a medley of fragmented signatures does not give a statistically significant result, since there are no complimentary sets in the databases (Figure 4).

Thus, there is incomplete detection of metabolic signatures in personal analysis. Meanwhile, their detection is not informative, since they most likely lie within the statistical norm. In addition, those metabolites that are outside of the norm cannot be analyzed, since they are a medley of incomplete signatures. Based on these described challenges, the current status of personal metabolomics may be summarized in the following points:

1. The accumulated scientific data show that metabolomic profiles contain comprehensive information about the organism state, which is supported by a wide variety of metabolic case–control studies. Metabolic profiles show high specificity and selectivity in classifying samples from healthy and sick patients (often exceeding 95%).
2. Today, there is no methodology (workflow) capable of personalized metabolomics. In fact, personalized metabolomics does not exist today. Measuring a limited number of metabolites is not relevant here, since it does not realize the essence of metabolomics—measuring an enormous number of metabolites for a panoramic study of biological samples.
3. The lack of personalized metabolomics is an algorithmic problem. Since metabolic profiles contain all of the necessary information for diagnosis (see point 1), there is only a need to extract it; however, there is no algorithm capable of doing this yet.
4. The creation of such an algorithm would revolutionize laboratory diagnostics. Hundreds of metabolic disease signatures would become measurable from a single drop of blood.

Therefore, it can be said that the accumulated data on metabolomics over the decades are useless for diagnostic purposes. It smacks of alarmism and repetition of history with other basic omics sciences. Genomic tests give results with a very low probability of assessed risks for most diseases, which essentially prevents them from leaving the field of wellness, thus entertaining gerontology and other pseudo-scientific applications. Moreover, it seemed that everything in the genome is written down and predetermined, but events significant for health are realized with a certain and, as a rule, very negligible probability (monogenic diseases are not considered here—genomic tests are not needed for their detection). The field of proteomics that followed genomics was also promising. As Friedrich Engels said, “Life is the mode of existence of protein bodies”. The high-throughput measurement of proteins could probably characterize the state of the life in the organism in detail. However, the euphoria passed; the “mountain has brought forth a mouse”. Proteomics is successfully used in scientific research, but there is practically no applied application in which its analytical ability to measure vast sets of proteins is realized. Metabolomics is the last of the basic omics sciences that thoroughly determines the molecular phenotype, and success in its applied application is most expected today. However, much more research is needed.

## 3. Conclusions

This article is not meant to be alarmist. A clear understanding of the problem is the first step in solving it. This article is intended to raise a discussion in this area, bring the problem to people, and consolidate scientists working in this area. Additionally, we are constantly faced with misunderstandings of scientists from related fields, doctors, managers of different levels, civil scientists, and even ordinary people related to why it is not possible to carry out a panoramic personal analysis using a modern mass spectrometer, powerful software, and databases with metabolic data describing the majority of diseases. Altogether, this prompted the writing of this article, which provides an answer to this question. The lack of personalized metabolomics is an algorithmic problem. Metabolic profiles contain all the necessary information for the diagnosis; there is only a need to extract it. However, there is no algorithm capable of doing this yet. The creation of such algorithms would solve this problem and revolutionize laboratory diagnostics. Hundreds of metabolic disease signatures would become measurable from a single drop of blood.

## Figures and Tables

**Table 1:** Data presented in metabolite databases.

Metabolite Database ^1^	Metabolic Data
Human Metabolome Database [11]	631 disease signatures
	808 human metabolic pathways
	abnormal concentrations of metabolites for 352 conditions
	110 sets based on organ, tissue, and subcellular localization
MetaboAnalyst [12]	**Disease signatures:**
	344 metabolite sets for human blood
	384 metabolite sets for human urine
	166 metabolite sets for human cerebral spinal fluid
	44 metabolite sets for human feces
	**Pathways:**
	99 metabolite sets based on normal human metabolic pathways
	84 human metabolic pathways
	461 metabolite sets based on drug pathways
	**Other types:**
	4598 metabolite sets based on their associations with single nucleotide polymorphism loci
	912 metabolic sets predicted to change in the case of dysfunctional enzymes
	73 metabolite sets based on organ, tissue, and subcellular localization
Small Molecule Pathway Database [13,14]	351 pathways (total number)
	113 disease pathways
	70 normal metabolic pathways
	168 drug action pathways

^1^ Data are presented for only three metabolite databases to show the amount of metabolomic data available for laboratory diagnosis—an overview of all databases is outside the scope of this article.

**Table 2:** Comparison of data processing in metabolomics case–control studies and personal metabolomics analysis.

Feature	Metabolomics Study	Personal Metabolomics Analysis
Design of study	Case-control type (group vs. group)	Sample vs. control set
Typical statistics	T-test (for normal distribution); Wilcoxon rank-sum test ^2^	Z-score; *p*-value ^1^
Detection of group-specific features	Yes	No
Biological insights are revealed	Yes (a vast amount of information related to disease, pathways, etc. is retrieved; these data fill the metabolic databases)	Impossible (the main challenge for personal metabolomics analysis)
Methods to reveal biological insights	Yes (e.g., metabolite set enrichment analysis)	None
Detection of biomarkers	Yes, but too rare	Impossible for detection new biomarkers, and easy for already discovered biomarkers
Detection of individual features	Yes, but considered as a noise useless for study purposes	Only prominent features or biomarkers
Medical application	Extremely difficult	Yes, but only to detect statistically significant features (e.g., metabolite biomarkers)

^1^ Z-score values can be converted into *p*-values in the case of a normal distribution. ^2^ The Wilcoxon rank-sum test (also called the Mann–Whitney–Wilcoxon, Mann–Whitney U test, or Wilcoxon–Mann–Whitney test) is a nonparametric test.

## Abbreviations

Laboratory-developed tests (LDTs), Clinical Laboratory Improvement Amendments (CLIA), dried blood spot (DBS).
